# Aberrant expression of Notch1/numb/snail signaling, an epithelial mesenchymal transition related pathway, in adenomyosis

**DOI:** 10.1186/s12958-015-0084-2

**Published:** 2015-08-26

**Authors:** Shasha Qi, Xingbo Zhao, Mingjiang Li, Xiaohui Zhang, Zhenzhen Lu, Chunrun Yang, Chunhua Zhang, Hui Zhang, Na Zhang

**Affiliations:** Department of Obstetrics and Gynecology, Shandong Provincial Hospital Affiliated to Shandong University, 324 Jingwu Road, Jinan, Shandong 250021 People’s Republic of China; Department of Anesthesiology and Surgery, Shandong Provincial Hospital Affiliated to Shandong University, 324 Jingwu Road, Jinan, Shandong 250021 People’s Republic of China

**Keywords:** *Adenomyosis*, *Epithelial Mesenchymal Transition*, *Notch1/Numb/Snail Signaling*, *Slug*

## Abstract

**Background:**

Epithelial mesenchymal transition (EMT) is involved in the pathogenesis of adenomyosis, and Notch signaling is crucial to EMT. The objective of this study was to explore Notch1/Numb/Snail signaling in adenomyosis.

**Methods:**

The expression levels of the members of the Notch1/Numb/Snail signaling cascade in normal endometria (proliferative phase: *n* = 15; secretory phase: *n* = 15; postmenopausal phase: *n* = 15) and adenomyotic endometria (proliferative phase: *n* = 15; secretory phase: *n* = 15) were determined by immunohistochemistry analysis.

**Results:**

We found that the expressions of Notch1 and the EMT-related proteins N-cadherin, Snail and Slug were upregulated in the ectopic endometrium of adenomyosis compared with normal endometrium. Numb, a negative regulator of Notch signaling, was significantly decreased in adenomyosis. In addition, reduced immunoexpression of E-cadherin was observed in adenomyosis.

**Conclusions:**

We conclude that Notch1/Numb/Snail signaling plays an important role in the pathogenesis and development of adenomyosis.

## Background

Adenomyosis is a prevalent gynaecologic benign condition of the uterus characterized by the presence of activated endometrium within the myometrium [1]. The disease leads to dysmenorrhea, dyspareunia, abnormal uterine bleeding, and infertility and significantly reduces the quality of life of women of reproductive age [2]. The best treatment for adenomyosis is still unclear, and the mechanism of thisdisease has not been determined.

Epithelial-mesenchymal transition (EMT) is a biological process during which epithelial cells lose their polarity and cell-cell contacts and acquire a migratory mesenchymal phenotype [3, 4]. The process of EMT is characterized by the loss of epithelial markers and the acquisition of mesenchymal markers [5]. The EMT plays a key role in tumour metastasis [6].

Migration and invasion are also considered key to the formation and progression of endometriosis [7]. Recent studies have shown that EMT-like processes may be involved in the pathogenesis of endometriosis [8, 9]. Weaker expression of epithelial markers and stronger expression of mesenchymal markers are present in ectopic epithelial cells of endometriotic lesions on peritoneal and ovarian tissues [8]. In the epithelial component of adenomyotic lesions, vimentin expression is up-regulated and E-cadherin expression is down-regulated compared to the eutopic endometrium [10].

The Notch signaling pathway is thought to be critical for the induction of EMT. The Notch family, which included four members, Notch1-4, is a family of single-pass transmembrane receptor proteins [11]. Mature Notch receptors are heterologous dimers, consisting of a large extracellular ligand binding domain, a single-pass transmembrane structure and a small cytoplasmic subunit (Notch intracellular domain, NICD) [12, 13].Transmembrane ligands of the DSL (Delta/Serrate/Lag2) family bind to Notch receptors, triggering heterodimer cleavage and release of the NICD. The NICD then enters the nucleus and modulates the transcription of downstream target genes, including EMT-related genes, such as Snail and Slug (also called Snail2) [13]. Snail and Slug can combine with the E-cadherin promoter to suppress its expression [14, 15]. Numb is an inhibitory regulator of Notch1 signaling that acts by promoting the ubiquitination and degradation of the Notch1 intracellular domain [16]. It has been reported that down-regulation or loss of Numb expression might be correlated with the genesis, development and enhancement of the invasion of multiple tumours [17, 18].

Notch signaling is involved in the process of EMT in a series of human tumours. Notch signaling can promote TGF-β1-induced EMT through the induction of Snai1 [19]. In various human cancer models, Jagged1-mediated notch signaling activation can elevate the expressions of Snail and Slug, resulting in the repression of E-cadherin [20]. In pancreatic cancer cells, over-expression of Notch-1 induces the EMT phenotype and increases cell growth, migration and invasion [21]. In lung cancer cells, inhibition of Notch signaling reverses the EMT process and, thus, enhances the therapeutic susceptibility of lung cancer cells [22]. In breast cancer cells, anti-human NICD monoclonal antibody can suppress the EMT process, inhibit cell growth and induce apoptosis [23]. Hypoxia-induced Notch signaling can affect EMT and migration of breast cancer cells by regulating the expression of Snail and Slug [24]. Moreover,Notch signaling can regulate the progression of metastatic hepatocellular carcinoma by regulating the expression of Snail and E-cadherin [25].

As shown above, Notch signaling cascades are crucial in the process of EMT. In the current study, we aimed to investigate the status of Notch1/Numb/Snail signaling in adenomyosis and to explore the possible role of this signaling pathway in the development and progression of this disease.

## Methods

### Materials and tissue collection

Rabbit anti-human Notch1 (NICD); mouse anti-human Numb, E-cadherin, N-cadherin, and Slug; and goat anti-human Snail primary antibodies were obtained from Abcam (Beverly, MA, USA). Goat anti-rabbit and goat anti-mouse HRP-conjugated secondary antibodies and Diaminobenzidine staining kits were obtained from ZSGB-BIO (Beijing, China).

Normal endometria were obtained from 45 women of reproductive age undergoing bilateral tubal ligation (proliferative phase: *n* = 15; secretory phase: *n* = 15; postmenopausal phase: *n* = 15). Adenomyotic lesions were obtained from 30 patients with adenomyosis undergoing hysterectomy or subtotal hysterectomy (proliferative phase: *n* = 15; secretory phase: *n* = 15) (Table 1). Normal endometria and adenomyosis tissues were collected during the operation.The diagnosis of adenomyosis was confirmed by histological examination. No patients received any hormonal therapy in three months prior to their surgery. Informed consent was obtained from all participants prior to the biopsy procedure, and the use of human tissues was approved by the institutional review board of Shandong Provincial Hospital Affiliated to Shandong University.

**Table 1:** Detailed information of patients

Proliferative phase		Secretory phase	
Ages	Dysmenorrhea	Ages	Dysmenorrhea
39	+	47	+
44	+	41	+
47	+	50	+
44	+	45	+
45	+	42	+
43	+	42	+
32	+	47	+
47	+	37	+
37	+	40	+
43	+	48	+
47	+	43	+
44	-	52	+
49	+	45	+
39	+	48	+
50	+	51	+

### Immunohistochemistry analysis

Immunohistochemistry analysis was performed on normal endometria and adenomyotic lesions. Fresh tissue samples were washed with PBS twice to remove blood. Then, they were fixed in 4 % paraformaldehyde for 24 h and embedded in paraffin. The samples were cut into 4 μm sections and mounted onto glass slides. Deparaffinized,rehydrated sections were incubated with 3 % H_2_O_2_ for 30 min to block endogenous peroxidase activity. Antigen retrieval was performed using a pressure-cooker for 90 seconds inEDTA buffer at pH 7.6. The sections were rinsed in PBS, blocked with 10 % normal goat serum or calf serum for 30 min,and then incubated with primary antibodies, including rabbit anti-human notch(diluted 1:200 in PBS), mouse anti-human numb (diluted 1:150 in PBS), E-cadherin (diluted 1:100 in PBS), N-cadherin (diluted 1:500 in PBS), Slug (diluted 1:150 in PBS) andgoat anti-human snail (diluted 1:100 in PBS)antibodies, overnight in a wet chamber at 4 °C. HRP-conjugated goat anti-rabbit or mouse IgG was used as the second antibody, as appropriate. HRP activity was detected by measuring the level of the substrate diaminobenzidine tetrahydrochloride (DAB) for 1 min. The sections were counterstained with haematoxylin before mounting. Sections incubated with non-immune serum instead of primary antibodies were used as the negative controls. The sections were observed under a Leica DM4000B microscope (Leica), and pictures were taken using the IM50 image analysis system (Leica).

The immunostaining was expressed as immunoscore-H-score, which was semiquantitative as a product of a quantity score and a staining intensity. The quantity score was estimated as follows: four random views are chosen and 100 cells were counted to get the percentages (Pi) of positively stained glandular epithelial cells. The staining intensity (I) of the glandular epithelial cells was estimated as follows: 0: negative; 1: weak staining; 2: moderate staining; and 3: strong staining. Two sections per sample were assessed by two observers. H-score = Pi (I + 1). All slides were evaluated for immunostaining without any knowledge of the clinical or pathological data.

### Statistical analysis

The data were statistically analysed by the two-tailed student’s unpaired t-test using SPSS 19.0 (SPSS Inc., Chicago, IL). Values are expressed as means ± SD. Differences between two groups were determined by the two-tailed student’s t-test. The level of statistical significance was set at *p* < 0.05.

## Results

### Notch1 expression was upregulated in adenomyosis

**Fig. 1:**
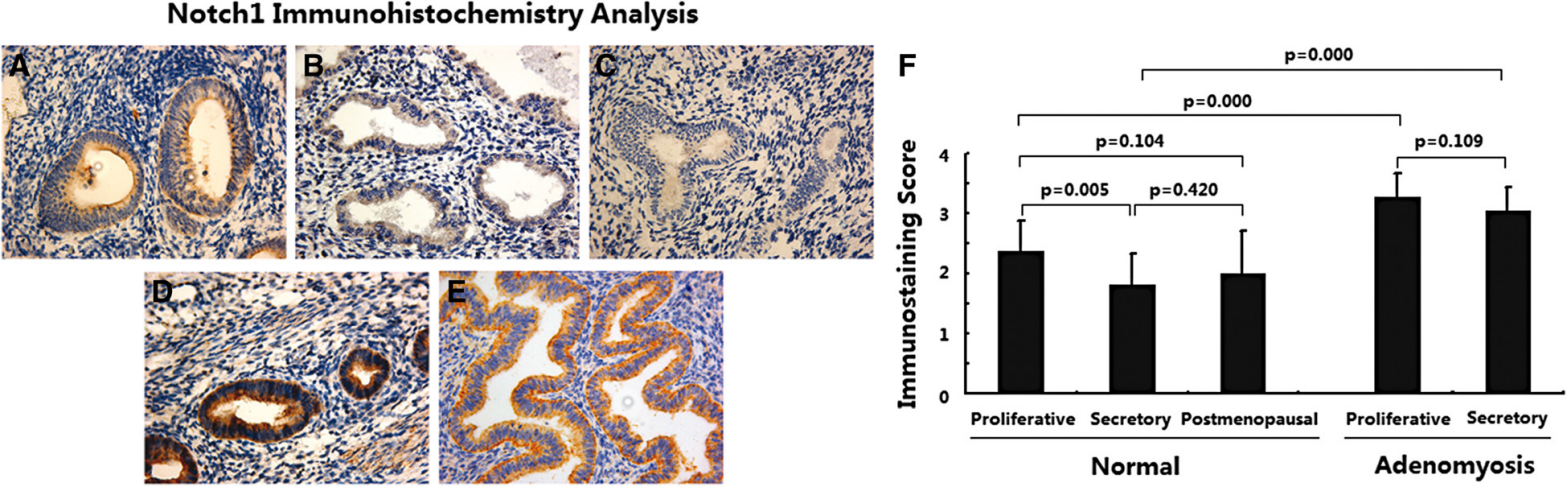
Immunoexpression of protein Notch1 in normal endometrium and ectopic endometrium from patients with adenomyosis. **a** normal endometrium of proliferative phase (*n* = 15); **b** normal endometrium of secretory phase (*n* = 15); **c** normal endometrium of postmenopausal phase (*n* = 15); **d** ectopic endometrium in adenomyosis of proliferative phase (*n* = 15); **e** ectopic endometrium in adenomyosis of secretory phase (*n* = 15); magnification: ×200; **f** Immunoscore of Notch1

The expression of Notch1 in normal endometria and ectopic endometria from adenomyotic lesions was determined using immunohistochemical analysis. As shown in Fig. 1, in normal endometria, the staining of Notch1 was weakly positive or positive and was concentrated in the cytoplasm of endometrial epithelial cells (Fig. 1, a-c). In stromal cells, the immunostaining of Notch1 was very weak. Endometria in the proliferative phase showed higher Notch1 expression than endometria in the secretory phase (Fig. 1a-b, *p* < 0.01). No significant difference in Notch1 expression was noted between endometria in the productive phase and endometria in the postmenopausal phase (Fig. 1a-c, *p* > 0.05).

In ectopic endometria of adenomyosis, the immunostaining of Notch1 was strongly positive and was also restricted to the cytoplasm of epithelial cells (Fig. 1d, e); weak immunostaining was observed in stromal cells. In addition, no significant difference in Notch1 expression was observed between ectopic endometria in the proliferative and secretory phases (Fig. 1d-e, *p* > 0.05). However, ectopic endometria of adenomyosis in both the proliferative and secretory phases showed significantly increased Notch1 expression compared to normal endometria (Fig. 1f, *p* < 0.01).

These data suggest that elevated Notch1 signaling is present in adenomyosis. Moreover, Notch1 expression was shown to change during the menstrual cycle in normal endometria but not in adenomyotic endometria.

### Numb expression was reduced in adenomyosis

**Fig. 2:**
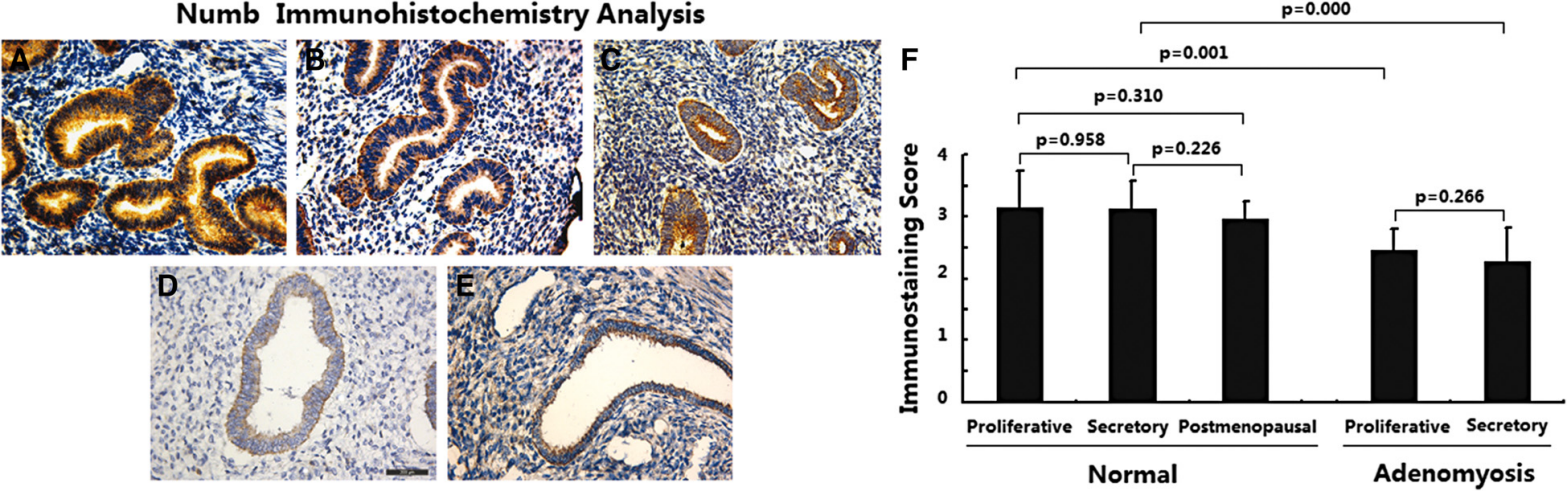
Immunoexpression of protein Numb in normal endometrium and ectopic endometrium from patients with adenomyosis. **a** normal endometrium of proliferative phase (*n* = 15); **b** normal endometrium of secretory phase (*n* = 15); **c** normal endometrium of postmenopausal phase (*n* = 15); **d** ectopic endometrium in adenomyosis of proliferative phase (*n* = 15); **e** ectopic endometrium in adenomyosis of secretory phase (*n* = 15); magnification: ×200; **f** Immunoscore of Numb

The expression of Numb in different endometria was determined by immunohistochemical analysis. As shown in Fig. 2, in normal endometria, the immunostaining of Numb was strongly positive and was most frequently distributed in the cytoplasm of endometrial epithelial cells; immunostaining in stromal cells was very weak. No significant difference in Numb expression was observed between endometria in the proliferative, secretory and postmenopausal phases (Fig. 2a-c, *p* > 0.05).

In ectopic endometria of adenomyosis, the immunostaining of Numb was weakly positive and was restricted to the cytoplasm of epithelial cells (Fig. 2d, e); weak immunostaining was observed in stromal cells. In addition, no significant difference in Numb expression was observed in ectopic endometria in the proliferative and secretory phases (Fig. 2d, e, *p* > 0.05). However, ectopic endometria of adenomyosis showed significantly decreased Numb expression in both the proliferative and secretory phases compared with normal endometria (Fig. 2d, e, *p* < 0.05).

These data suggest that Numb expression did not change during the menstrual cycles in either normal endometria or adenomyotic endometria and that Numb expression was lost in adenomyosis.

### Snail expression was increased in adenomyosis

**Fig. 3:**
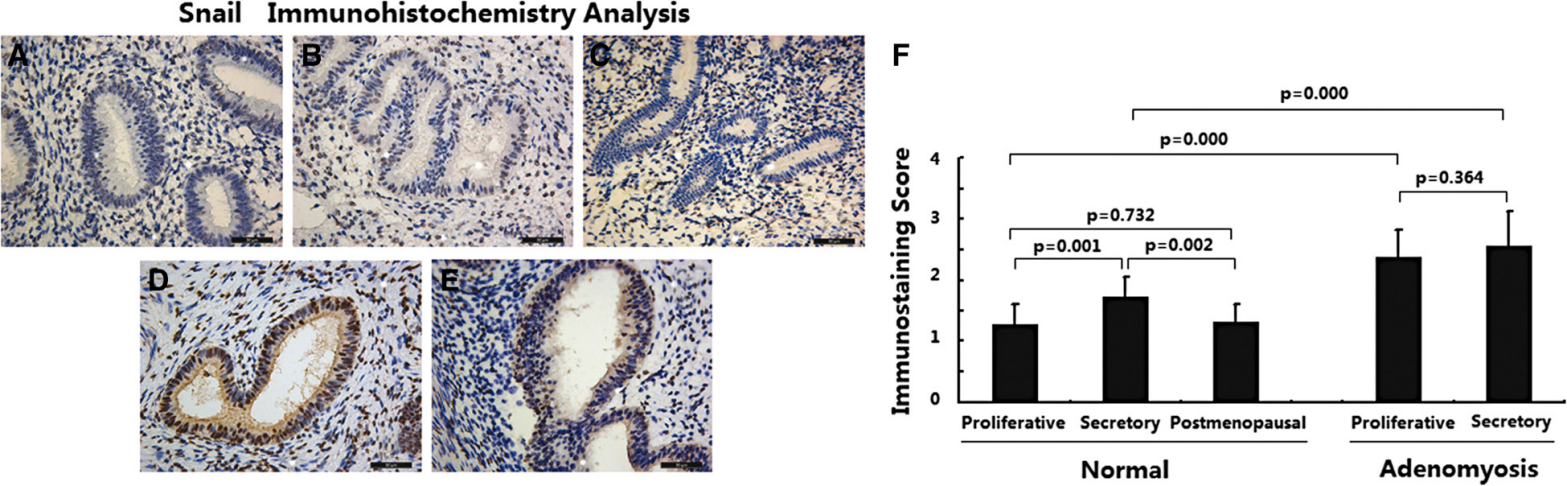
Immunoexpression of protein Snail in normal endometrium and ectopic endometrium from patients with adenomyosis. **a** normal endometrium of proliferative phase (*n* = 15); **b** normal endometrium of secretory phase (*n* = 15); **c** normal endometrium of postmenopausal phase (*n* = 15); **d** ectopic endometrium in adenomyosis of proliferative phase (*n* = 15); **e** ectopic endometrium in adenomyosis of secretory phase (*n* = 15); magnification: ×200; **f** Immunoscore of Snail

The expression of Snail in different endometria was determined using immunohistochemical analysis. As shown in Fig. 3, in normal endometria, the immunostaining of Snail was negative or weakly positive and was restricted to the nucleus of endometrial glandular epithelial cells; immunostaining in stromal cells was very weak. Endometria in the proliferative and postmenopausal phases showed decreased Snail expression compared with endometria in thesecretory phase (Fig. 3a-c, f, *p* < 0.01). No significant difference in Snail expression was observed between endometria in the productive and postmenopausal phases (Fig. 3a-c, f, *p* > 0.05).

In ectopic endometria of adenomyosis, the immunostaining of Snail was strongly positive and was restricted to the nucleus of epithelial cells (Fig. 3d, e); weaker immunostaining was observed in stromal cells. No significant difference in Snail expression was observed between ectopic endometria in the proliferative and secretory phases (Fig. 3d-f, *p* > 0.05); however, ectopic endometria of adenomyosis showed significantly increased Snail expression in both the proliferative and secretory phases compared with normal endometria(Fig. 3a-f, *p* < 0.01).

These data suggest that Snail expression is elevated in adenomyosis. In addition, Snail expression changed during the menstrual cycle in normal endometriabut not in adenomyotic endometria.

### Slug expression was upregulated in adenomyosis

**Fig. 4:**
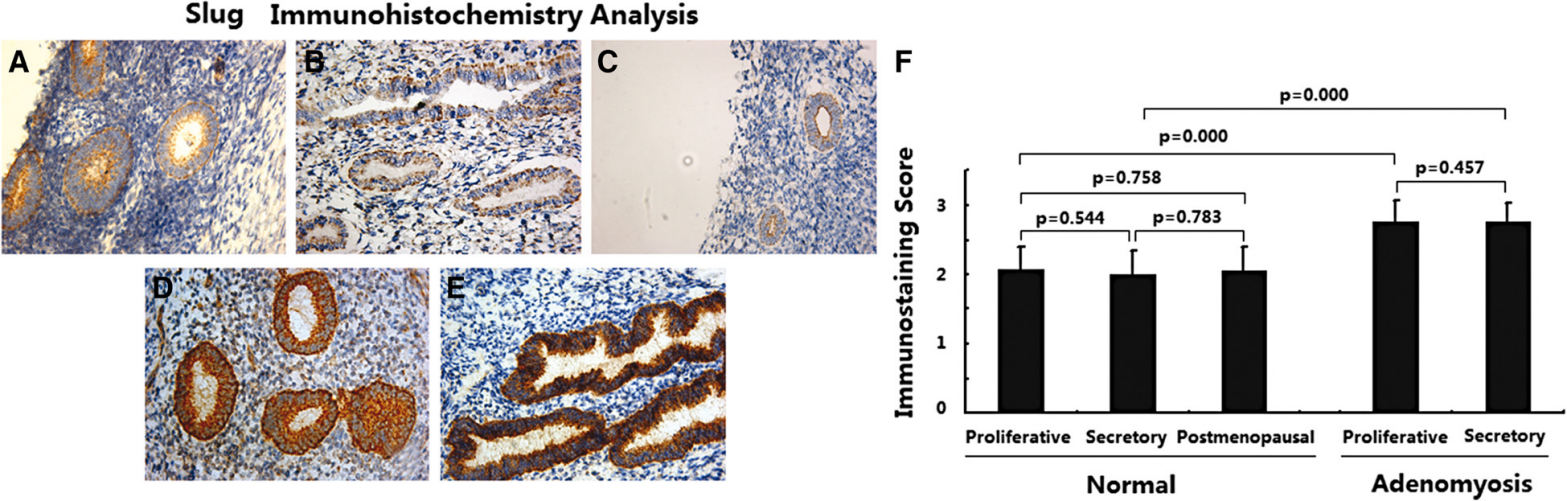
Immunoexpression of protein Slug in normal endometrium and ectopic endometrium from patients with adenomyosis. **a** normal endometrium of proliferative phase (*n* = 15); **b** normal endometrium of secretory phase (*n* = 15); **c** normal endometrium of postmenopausal phase (*n* = 15); **d** ectopic endometrium in adenomyosis of proliferative phase (*n* = 15); **e** ectopic endometrium in adenomyosis of secretory phase (*n* = 15); magnification: ×200; **f** Immunoscore of Slug

The expression of Slug in different endometria was determined by immunohistochemical analysis. As shown in Fig. 4, in normal endometria, the immunostaining of Slug was weakly positive or positive and was usually distributed in the cell membrane of endometrial epithelial cells; immunostaining in stromal cells was very weak. No significant difference in Slug expression was observed between endometria in the proliferative, secretory and postmenopausal stages (Fig. 4a-c, *p* > 0.05).

In ectopic endometria of adenomyosis, the immunostaining of Slug was also strongly positive and was restricted to the cell membrane of epithelial cells; weaker immunostaining was observed in stromal cells. No significant difference in Slug expression was observed between ectopic endometria in the proliferative and secretory phases (Fig. 4d-f, *p* > 0.05); however, ectopic endometria of adenomyosis in both the proliferative and secretory phases showed significantly increased Slug expression compared with normal endometria (Fig. 4d-f, *p* < 0.01).

These data suggest that Slug expression did not change during the menstrual cycle in either normal endometria or adenomyotic endometria and that Slug expression was increased in adenomyosis.

### N-cadherin expression was upregulated in adenomyosis

**Fig. 5:**
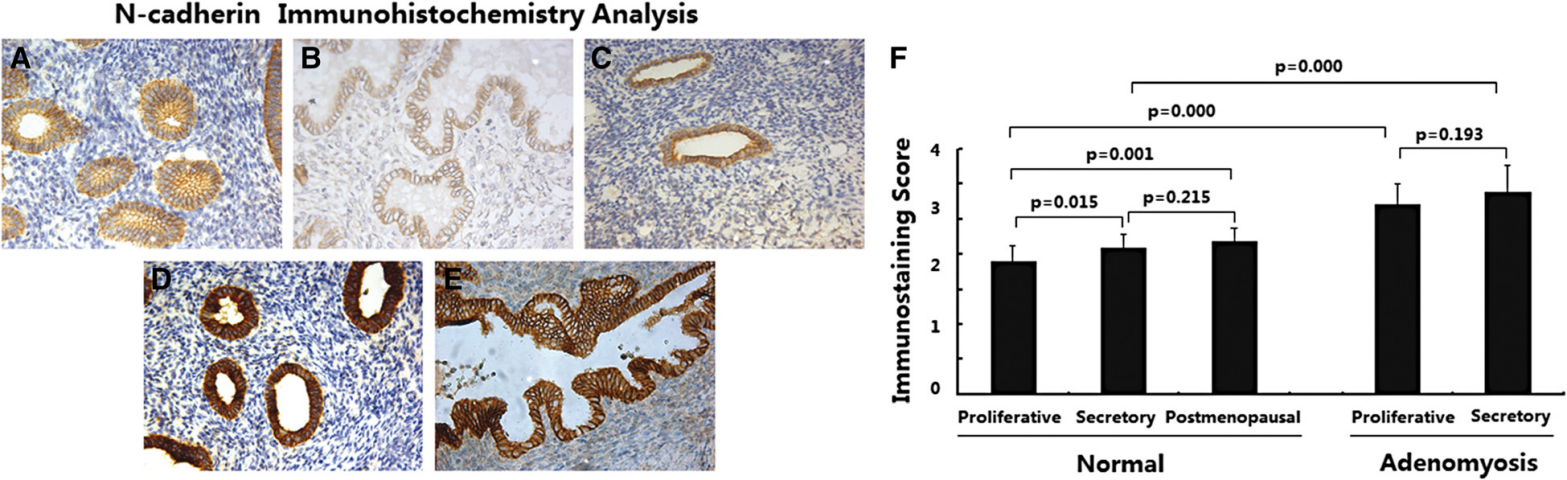
Immunoexpression of protein N-Cadherin in normal endometrium and ectopic endometrium from patients with adenomyosis. **a** normal endometrium of proliferative phase (*n* = 15); **b** normal endometrium of secretory phase (*n* = 15); c normal endometrium of postmenopausal phase (*n* = 15); **d** ectopic endometrium in adenomyosis of proliferative phase (*n* = 15); **e** ectopic endometrium in adenomyosis of secretory phase (*n* = 15); magnification: ×200; **f** Immunoscore of N-Cadherin

The expression of N-cadherin in different endometria was determined by immunohistochemical analysis. As shown in Fig. 5, in normal endometria, the immunostaining of N-Cadherin was weakly positive or positive and was usually distributed in the membrane of endometrial epithelial cells; immunostaining in stromal cells was very weak. Endometria in the secretory and postmenopausal phases showed higher N-cadherin expression than endometria in the proliferative phase (Fig. 5a-b, *p* < 0.05). No significant difference in N-cadherin expression was observed between endometria in the secretory and postmenopausal phases (Fig. 5a-c, *p* > 0.05).

In ectopic endometria of adenomyosis, the immunostaining of N-cadherin was strongly positive and was restricted to the cytoplasm of epithelial cells (Fig. 5d, e); weak immunostaining was observed in stromal cells. No significant difference inN-cadherin expression was observed between ectopic endometria in the proliferative and secretory phases (Fig. 5d-f, *p* > 0.05); however, ectopic endometria of adenomyosis in both the proliferative and secretory phases showed significantly increased N-cadherin expression compared with normal endometria (Fig. 5f, *p* < 0.01).

These data suggest that elevated N-cadherin expression is present in adenomyosis. N-cadherin expression changed during the menstrual cycles in normal endometria but not in adenomyotic endometria.

### E-cadherin expression was downregulated in adenomyosis

**Fig. 6:**
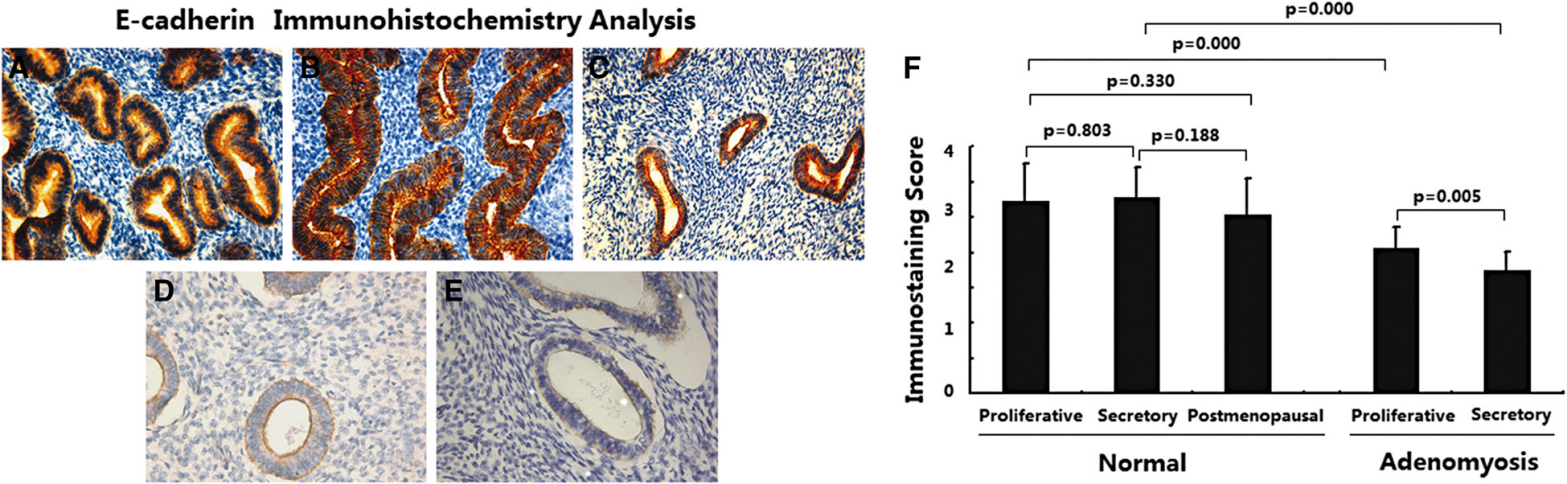
Immunoexpression of protein E-Cadherin in normal endometrium and ectopic endometrium from patients with adenomyosis. **a** normal endometrium of proliferative phase (*n* = 15); **b** normal endometrium of secretory phase (*n* = 15); c normal endometrium of postmenopausal phase (*n* = 15); **d** ectopic endometrium in adenomyosis of proliferative phase (*n* = 15); **e** ectopic endometrium in adenomyosis of secretory phase (*n* = 15); magnification: ×200; **f** Immunoscore of E-Cadherin

The expression ofE-cadherinin different endometria was determined by immunohistochemical analysis. As shown in Fig. 6, in normal endometria, the immunostaining of E-cadherin was strongly positive and was usually distributed in the membrane of endometrial epithelial cells; immunostaining in stromal cells was very weak. No significant difference of E-cadherin expression was observed between the endometria in different phases (Fig. 6a-c, f, *p* > 0.05).

In ectopic endometria of adenomyosis, the immunostaining of E-cadherin was weakly positive and was restricted to the membrane of epithelial cells (Fig. 6d, e); weak immunostaining was observed in stromal cells. Ectopic endometria in the proliferative phase showed higher E-cadherin expression than ectopic endometria in the secretive phase (Fig. 6d-f, *p* < 0.01). In addition, ectopic endometria of adenomyosis showed significantly decreased E-cadherin expression in both the proliferative and secretory phases compared with normal endometria (Fig. 6d-f, *p* < 0.05).

These data indicate that reduced E-cadherin expression is present in adenomyosis.

## Discussion

Adenomyosis is adisease that exists independent from endometriosis [2]. The main pathological changes of adenomyosis are the invasion of functional endometrial glands and stroma into the myometrium and the growth of ectopic glands or stroma in the myometrium and/or local hyperplasia [1]. Although adenomyosis is a benign disease, it exhibits a series of biological behaviours that are similar to those of malignant tumours, including adhesion, invasion, and implantation [26]. EMT is a process during which epithelial cells undergo phenotypic transformation into mesenchymal cells [5]. A great dealof evidence indicates that EMT is associated with the invasive and migratory behaviours of cancer cells, which enhance the metastatic ability of these cells [6, 7]. Chen et al. reported that EMT markers are aberrantly expressed in adenomyosis [10]. In the current study, we found that the EMT-related Notch1/Numb/Snail signaling pathway plays an important role in the pathogenesis of adenomyosis.

Notch signalingis involved in cell proliferation, survival, apoptosis, and differentiation, and alterations in Notch signaling are linked to tumourigenesis [27]. Notch activation in endothelial cells results in the down-regulation of endothelial markers and the up-regulation of mesenchymal markers [28]. In the EMT process, Notch signalingcrosstalks with multiple transcription and growth factors that are relevant to EMT, such as Snail, Slug, TGF-β, FGF, and PDGF [29, 30].

In human endometrium, Notch1-3 are expressed not only stromal cells but also in glandular epithelial cells, and Jagged and DDL4 are mainly expressed in glandular epithelial cells [31]. Mori et al. reported that the expression of Notch1 in the endometrium is higher during the proliferative phase than the secretory phase and is lowest during the postmenopausal phase [32]. In contrast, Cobellis et al. found that the expressions of Notch1 and Jagged1 increased from the proliferative phase to the secretory phase [33]. Notch1 plays an important role in the differentiation and decidualization of endometrial stromal cells [34]. During this process, the expression of Notch1 is down-regulated and the expression of Numb is up-regulated [35]. In endometrial carcinoma, the expressions of Notch, Jagged1, and DLL4 are significantly increased and are related to the stage and prognosis of the disease, and blockage of the Notch signaling pathway significantly inhibits the growth and invasion of endometrial adenocarcinoma cells [32, 36]. In addition, blockage of Notch signaling induces apoptosis in Ishikawa cells [37], while increased oestrogen promotes the growth of Ishikawa cells by activating the Notch signaling pathway [38]. In the current study, elevated Notch1 expression was noted in adenomyosis, suggesting its significant role in this disease. We found that endometrium in the proliferative phase showed higher Notch1 expression than that in the secretory phase and that endometrium in the postmenopausal phase showed the lowest level of Notch1 expression. These data are consistent with those of the study by Mori et al. Moreover, in our study, Notch1 expression changed during the menstrual cycle in normal endometria but not in adenomyotic endometria. These data indicate that of Notch1 expression in normal endometrium is regulated by hormones and that this hormonal sensitivity is aberrant in adenomyosis.

Numb protein was first observed in *Drosophila* [39] and is thought to be a cell fate determinant that acts by regulating cell division, adhesion, and migration [40] In mammals, Numb protein inhibits Notch signaling by promoting the ubiquitination of the Notch1 receptor and the degradation of Notch1 intracellular domain (NICD) [16]. Numb is considered a tumour suppressor [41] in various carcinomas, including breast cancer [42] and salivary gland carcinomas [43]. However,Numb overexpression has been observed in astrocytomas [44] and cervical squamous carcinoma cells [45], implying that Numb may be oncogene in these diseases. In the current study, we investigated Numb expression in normal endometrium and adenomyotic endometrium, and we found that Numb expression did not change during the menstrual cycle in either normal endometria or adenomyotic endometria, indicating that Numb expression is hormonally independent. In addition, the loss of Numb expression was noted in adenomyosis, demonstrating that aberrant negative regulation of Numb may be involved in the genesis and development of adenomyosis. To our knowledge, the current study is the first to explore the role of Numb in adenomyosis.

EMT is activated by a number of transcription factors, including Snail, Slug, and Twist, and also by the repression of E-cadherin expression [46]. Snail and Slug have been reported to be associated with tumour cell migration, invasion, and metastasis. Snail was first discovered in Drosophila as a zinc-finger transcription factor and has since been proven to be a key regulator of EMT [47]. Snail also represses E-cadherin transcription by binding to the E-box site in the promoter of E-cadherin [48]. The role of Snail in EMT regulation has been reported in multiple carcinoma types, including breast carcinoma, ovarian carcinoma, etc. [48, 49]. Slug, which belongs to the Slug family of zing-finger transcription factors, also plays a major role in EMT during embryonic development and metastasis of various cancers by inhibiting E-cadherin [50]. In ovarian carcinoma cells, increased expression of Snail and Slug directly lead to cisplatin resistance [51] and promote the EMT process by activating theβ-Catenin–T-Cell Factor-4-dependent expression of transforming growth factor-β3 [52]. Functional knockdown of Snail and Slug was shown to significantly decrease the tumourigenicity and metastatic behaviour of squamous carcinoma cells [53]. In the current study, we discovered that Snail and Slug were upregulated in adenomyosis, indicating the possible role of Snail/Slug-associated EMT in the pathogenesis and development of adenomyosis. In addition, Snail expression changed during the menstrual cycle in normal endometria, but Slug expression did not change during the menstrual cycle in either normal endometria or adenomyotic endometria. Furthermore, the menstrual changes in Snail expression were absent in adenomyosis, suggesting the decreased hormonal sensitivity of the ectopic endometrium of adenomyosis.

N-cadherin is another EMT marker. A switch from expression of E-cadherin to expression of N-cadherin is frequently observed in many aggressive cancers [27]. N-cadherin stimulates the upregulation of Snail and Slug in a FGFR-dependent manner [54]. N-cadherin-mediated cell adhesion accelerates cell migration in a three-dimensional matrix [55]. In adenomyosis, we found that N-cadherin was up-regulated in ectopic epithelial cells, indicating the important role of N-cadherin in this disease. Moreover, N-cadherin expression changed during the menstrual cycle in normal endometrium but not in adenomyotic endometrium. These data suggest decreased hormonal sensitivity in adenomyosis.

One of the most common features of EMT is the loss of E-cadherin expression [27]. During the EMT process, epithelial cells undergo a phenotypic switch to the mesenchymal phenotype, which leads to the loss of cell-cell adhesion, alternation of polarity, modulation of the cytoskeletal systems, and a switch of expression from keratin to vimentin [5]. Inhibition of Snail may stimulate the re-expression of E-cadherin and other epithelial markers in metastatic tissues, where higher expression of E-cadherin and epithelial characteristics may contribute to increased survival and proliferation [56]. In prostate cancer, E-cadherin and Snail levels can be measured to assess disease prognosis and can be used as therapeutic targets to prevent metastatic progression [57].A previous study reported that E-cadherin expression was decreased in the uterus of mice and in human adenomyotic lesions [56]. Consistent with the results of Shih et al., in the current study, the expression of E-cadherin was significantly reduced in ectopic epithelial cells of adenomyotic endometrium. In addition, E-cadherin expression showed no hormonal dependence in normal endometrium, while higher E-cadherin expression was noted in adenomyotic endometrium in the proliferative phase compared than in adenomyotic endometrium in the secretory phase.

## Conclusion

**Fig. 7:**
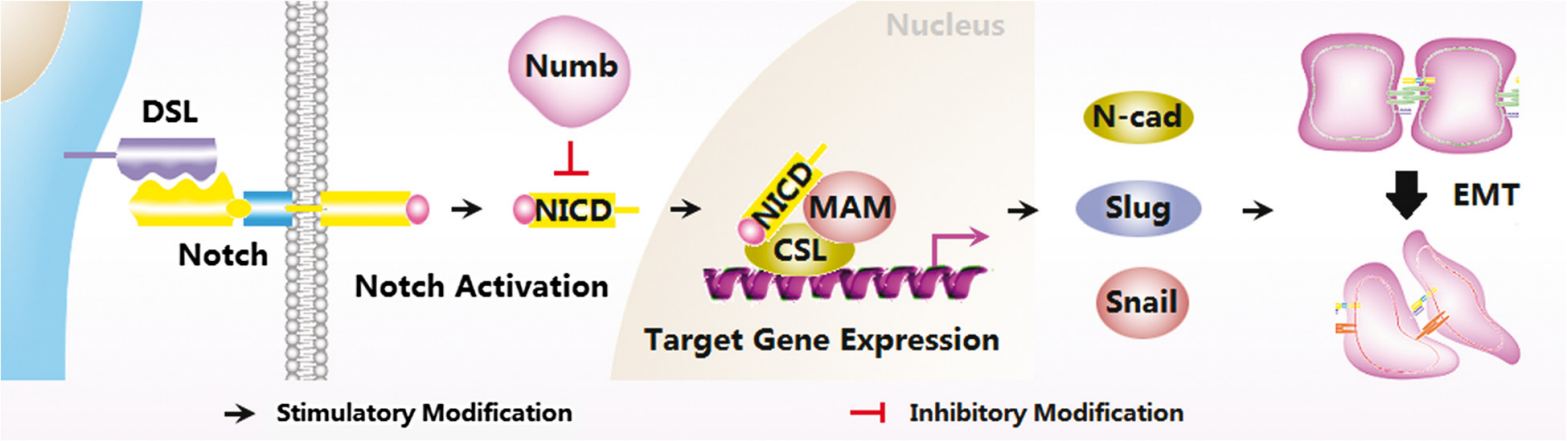
Schematic representation of Notch1/Numb/Snail signaling-induced EMT in adenomyosis

In conclusion, our data demonstrate the possible involvement of Notch1/Snail/Numb signaling in the pathogenesis and development of adenomyosis (Fig. 7). The current study may provide new insight into the diagnosis and treatment of adenomyosis.However, the main limitation of this study is that we only examined the expression and location of Notch1/Numb/Snail signaling by immunohistochemistry. In our next study, our team will examine the involvement of Notch1 signaling in adenomyosis using multiple experimental techniques.
